# Identification of Kunitz-Type Inhibitor Gene Family of *Populus yunnanensis* Reveals a Stress Tolerance Function in Inverted Cuttings

**DOI:** 10.3390/ijms26010188

**Published:** 2024-12-29

**Authors:** Haiyang Guo, Shaojie Ma, Xiaolin Zhang, Rong Xu, Cai Wang, Shihai Zhang, Lihong Zhao, Dan Li, Dan Zong

**Affiliations:** 1 Key Laboratory for Forest Genetics and Tree Improvement and Propagation in University of Yunnan Province, Southwest Forestry University, Kunming 650224, China; ghy10@swfu.edu.cn (H.G.); msj1013@swfu.edu.cn (S.M.); xl27@swfu.edu.cn (X.Z.); xxdr@swfu.edu.cn (R.X.); w413220@swfu.edu.cn (C.W.); zhangshihai@swfu.edu.cn (S.Z.); zhaolihong@swfu.edu.cn (L.Z.); 2 Key Laboratory for Forest Resources Conservation and Utilization in the Southwest Mountains of China, Ministry of Education, Southwest Forestry University, Kunming 650224, China; lidan@swfu.edu.cn; 3 Yunnan Provincial Key Laboratory for Conservation and Utilization of Inforest Resource, Southwest Forestry University, Kunming 650224, China

**Keywords:** *Populus yunnanensis*, *KTI* gene family, inverted cuttings, transcriptome

## Abstract

Plant protease inhibitors are a ubiquitous feature of plant species and exert a substantial influence on plant stress responses. However, the *KTI* (Kunitz trypsin inhibitor) family responding to abiotic stress has not been fully characterized in *Populus yunnanensis*. In this study, we conducted a genome-wide study of the *KTI* family and analyzed their gene structure, gene duplication, conserved motifs, cis-acting elements, and response to stress treatment. A total of 29 *KTI*s were identified in the *P. yunnanensis* genome. Based on phylogenetic analysis, the Py*KTI*s were divided into four groups (1,2, 3, and 4). Promoter sequence analysis showed that the Py*KTI*s contain many cis-acting elements related to light, plant growth, hormone, and stress responses, indicating that Py*KTI*s are widely involved in various biological regulatory processes. RNA sequencing and real-time quantitative polymerase chain reaction analysis showed that *KTI* genes were differentially expressed under the inverted cutting stress of *P. yunnanensis*. Transcriptome analysis of *P. yunnanensis* leaves revealed that *PyKTI16*, *PyKTI18*, and *PyKTI19* were highly upregulated after inverted cutting. Through the GEO query of *Populus transcriptome* data, *KTI* genes played a positive defense role in MeJa, drought, time series, and pathogen stress. This study provided comprehensive information for the *KTI* family in *P. yunnanensis*, which should be helpful for the functional characterization of *P. yunnanensis KTI* genes in the future.

## 1. Introduction

Proteases play vital roles in many cellular processes, such as digestion, blood clotting, immune response, and cellular signaling. They are involved in protein turnover and regulation, ensuring proper functioning and homeostasis within living organisms. These activities of proteases are controlled by protease inhibitors [1]. Protease inhibitors are molecules that inhibit the action of proteases [2]. In the past couple of decades, the number of studies on plant proteases and their proteinaceous inhibitors has been interestingly increased. The Kunitz trypsin inhibitor (*KTI*) is a protease inhibitor that contains a unique Kunitz domain and specifically inhibits trypsin. According to the classification by Rawlings et al. (2004), *KTI* belongs to the I3 inhibitor family, primarily functioning in the regulation of extracellular and intracellular target protease activities [3,4]. It is involved in physiological processes such as plant cell growth, cell cycle control, apoptosis, protein degradation and transport, stress response, and developmental growth [5].

KTI, which was first isolated from soybeans by Kunitz in 1945 [6], belongs to the family of protease inhibitors (PIs) and exhibits the ability to inhibit serine proteases. It has been postulated that *KTI*s play a crucial role in conferring resistance against herbivores and pathogens in numerous plant species. Notably, a KTI protein derived from the roots of *Pseudostellaria heterophylla* exhibited remarkable inhibitory activity against *Fusarium oxysporum* [7]. Multiple Kunitz trypsin inhibitors have been identified in *Solanum tuberosum* tubers, and research has revealed their potential as antifungal and antimicrobial agents [8]. In *Arabidopsis thaliana*, the silencing of *AtKTI1* resulted in reduced resistance to *Pseudomonas syringae* pv tomato DC3000, whereas the overexpression of *AtKTI1* enhanced resistance against the pathogen [9]. Additionally, studies have demonstrated that *NtKTI1* plays a crucial role in conferring resistance to various pathogens, including *Rhizoctonia solani*, Rhizopus nigricans, and *Phytophthora parasitica* var. nicotianae, in *Nicotiana tabacum* [10]. These findings highlight the importance of KTIs in plant defense mechanisms against diverse pathogens and emphasize their potential applications in crop protection strategies. The involvement of *AtKTI4* and *AtKTI5* in defense against spider mites was observed, with elevated levels of serine protease and cysteine protease inhibitor activity detected in leaf protein extracts upon overexpression of these two genes in *Nicotiana benthamiana* [11]. In *Passiflora edulis* fruit, a total of seven Kunitz trypsin inhibitors (*KTI*s) have been characterized, demonstrating their role in conferring resistance against *Diatraea saccharalis* [12]. This discovery sheds light on the potential of *KTI*s in contributing to the plant’s defense mechanisms against insect herbivory and highlights their significance in the context of plant–insect interactions.

*Populus yunnanensis*, a dioecious poplar species endemic to the southwestern region of China, exhibits a wide distribution in areas characterized by low latitudes and high elevations [13]. Due to its rapid growth and ability for easy-cutting propagation, *P. yunnanensis* holds a prominent position as a dominant tree species in both forestry production and environmental conservation efforts. Its suitability for various applications makes it a valuable resource in terms of both economic and ecological significance [14]. Upon conducting cutting experiments, we serendipitously observed that inverted cuttings of *P. yunnanensis* not only survived but also exhibited the ability to develop into fully formed trees [15]. This phenomenon was facilitated by the rooting process occurring at the original morphological apex and subsequent sprouting at the base, effectively sustaining the survival and growth of the cuttings. These findings provide valuable insights into the remarkable regenerative capabilities of *P. yunnanensis* and may have implications for propagation and cultivation practices. Subsequently, the inverted cuttings of *P. yunnanensis* exhibited delayed sprouting and a bending upward growth pattern. Eventually, lateral branches that emerged from the base bud of the stem exhibited strong growth, albeit slightly less vigorous than those of the upright branches [15,16]. The levels of many hormones in *P. yunnanensis*, including auxins (IAA), cytokinins (CTK), gibberellin (GAs), ethylene(ET), and brassinosteroids (BRs), underwent large changes in the inverted cuttings [16]. The growth response observed in plants subjected to inversion presents an intriguing area of research for investigating the mechanisms underlying this phenomenon and its potential applications in horticultural and forestry practices.

This research is the first to identify, analyze, and function the *KTI* gene family in *P. yunnanensis* at the whole-genome level. The expression of the *KTI* gene was analyzed in combination with the transcriptome of *P. yunnanensis* treated with inversion so as to provide basic research on the survival mechanism of inversion.

## 2. Results

### 2.1. Identification and Phylogenetic Tree of the PyunKTI Genes Family in Populus

**Figure 1:**
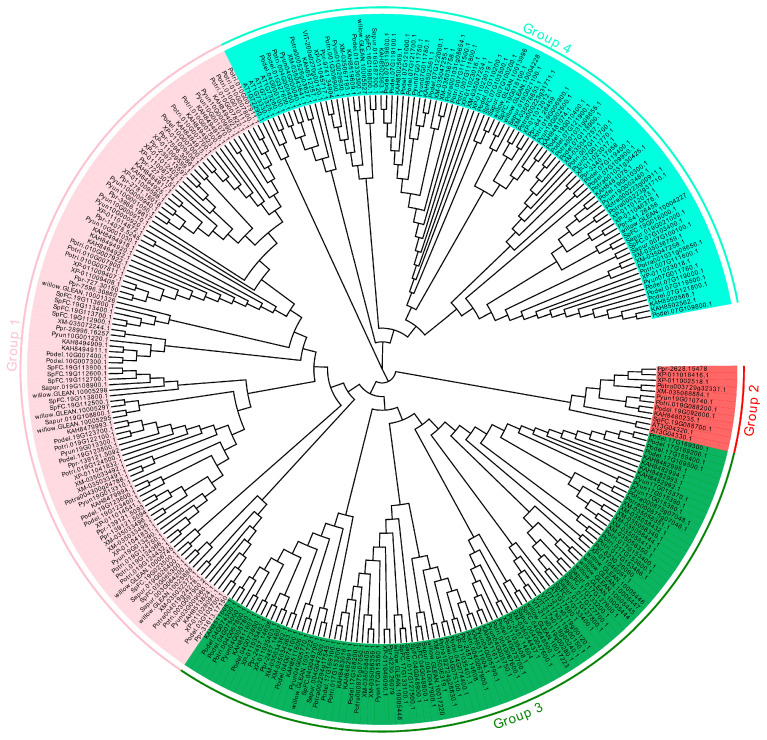
Phylogenetic relationship of *KTI* gene family. The MEGA 11 with the neighbor-joining method was used to conduct the phylogenetic tree. Different background colors represented different groups of the *KTI* gene family.

**Figure 2:**
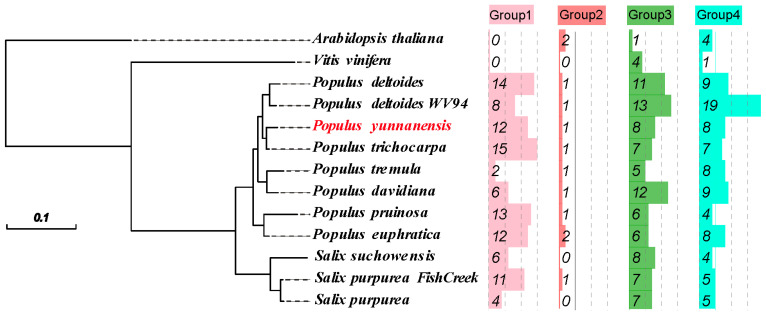
Phylogeny during the evolution of angiosperms. Different colors represent the proportion of each species in the four groups.

In the genomes of *A. thaliana*, *V. vinifera*, *Salix*, and *Populus*, 301 sequences were confirmed as Kunitz genes, including 29 in *Populus yunnanensis*, 30 in *Populus trichocarpa*, 41 in *Populus deltoides* WV94, 35 in *Populus deltoides*, 16 in *Populus tremula*, 28 in *Populus davidiana*, 28 in *Populus euphratica*, 24 in *Populus pruinosa*, 16 in *Salix purpurea*, 24 in *Salix purpurea,* 18 in *Salix suchowensis*, 5 in *V. vinifera*, and 7 in *A. thaliana* (Tables S1 and S2). Phylogenetic trees were constructed using the 301 *KTI*s from 13 species, and four groups were divided (Figure 1). Our 301 *KTI*s were clustered into four groups as follows: 95 within group 1, 12 within group 2, 103 within group 3, and 91 within group 4 (Figure 2). The *KTI*s of *Salix* and *Populus* plants were clustered, indicating that the *KTI* sequences were conserved among Salicaceae.

### 2.2. Characterization of the PyunKTIs Gene Family in P. yunnanensis

The Kunitz genes in *P. yunnanensis* were named *PyunKTI1~29* (Table 1). Bioinformatics analysis showed that the amino acid lengths of *PyunKTI1~29* were 183–259. Most of the KTI proteins (75.86%) were predicted to be acidic, and basic protein (24.14%) was distributed in group 3 and group 4. The instability index (II) and GRAVY of the 29 *PyunKTIs* proteins were greater than 20 and less than 0.5, respectively. In addition, the prediction of subcellular localization showed that all *PyunKTIs* proteins were located in the vacuole.

The signal peptides prediction of the 29 *PyunKTI* proteins almost belongs to the “standard” secretory signal peptides transported by the sec translocon and cleaved by Signal Peptidase I (SP I) and were differentiated into four different groups: Group 1 has SP positions at the 1 bp–26 bp/27 bp/28 bp amino acid site. Group 2 does not have SP positions. Group 3 has SP positions at 1–24/25, and, specifically, *PyunKTI4* has positions at 1–19. Group 4 has all SP positions at 1–21 (Table S3).

The hydrophobicity analysis of 29 *KTI* genes using Prot-Scale was used to further confirm more precise regions, combined with the prediction results of TMHMM for the transmembrane region (Table S4). In general, the transmembrane region of the protein is hydrophobic, while the part outside the membrane is hydrophilic [17]. The results showed that the predicted results of TMHMM and Prot-Scale were consistent, and the results of groups 1, 3, and 4 were less different. The amino acids from 1 to 30 had obvious hydrophobic characteristics and were in the transmembrane region, which was predicted to be the signal peptide sequence. In group 2, the amino acid sequence of *PyunKTI26* was hydrophilic from 1 to 40 and hydrophobic at sequence sites 40 to 60, possibly a transmembrane helix region, but not a signal peptide. These findings suggest that the characteristic features of PyunKTI proteins may include instability, hydrophilicity, and nuclear localization.

**Figure 3:**
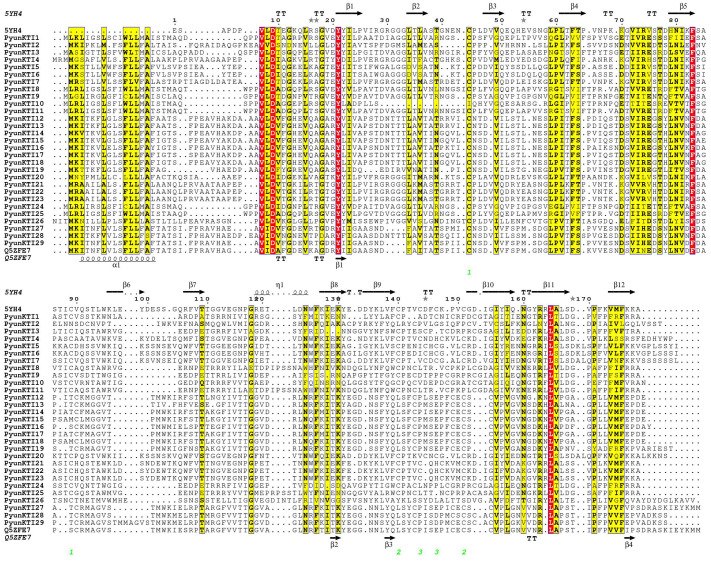
Sequence alignment of functional sites of PDB and PyunKTI proteins of *V. vinifera* (5YH4) and *Populus* (Q5ZFE7) homologous proteins. A red background indicates complete amino acid sequence identity at the position; a yellow background indicates higher similarity.

PDB files of *V. vinifera* (5YH4) and *Populus* (Q5ZFE7) homologous proteins with the best alignment results were downloaded from the PDB database. ESPript3.0 was used to refine the protein alignment file and add a secondary structure. The secondary structures of the 29 PyunKTI proteins were composed of an α-helix (6.51–20.79%), extended strand (28.64–37.07%), beta turn (4.43–8.91%), and random coil (37.84–53.59%) (Table S5 and Figure S1). In protein alignment files, the α-helix was mainly in the front end of the protein alignment sequence, the grape PDB file did not contain an α-helix, and the beta turn was predicted to be 4 in the *Populus* PDB file, but 12 in *V. vinifera* (Figure 3).

Protein tertiary structure prediction of the 29 *PyunKTI* proteins with Swiss-model homologous modeling. Except for *PyunKTI2*, the other *PyunKTI* prediction results were modeled by miraculin-like protein from *V. vinifera* (5yh4.1). The global model quality estimate ranged from 0.44 to 0.78, the identity ranged from 30.91% to 72.73%, and the accuracy of the model reached more than 80%. The prediction results could be used to find functional sites and predict functional relationships (Table S6).

### 2.3. Gene Structures and Conserved Motifs of the PyunKTI Genes Family

**Figure 4:**
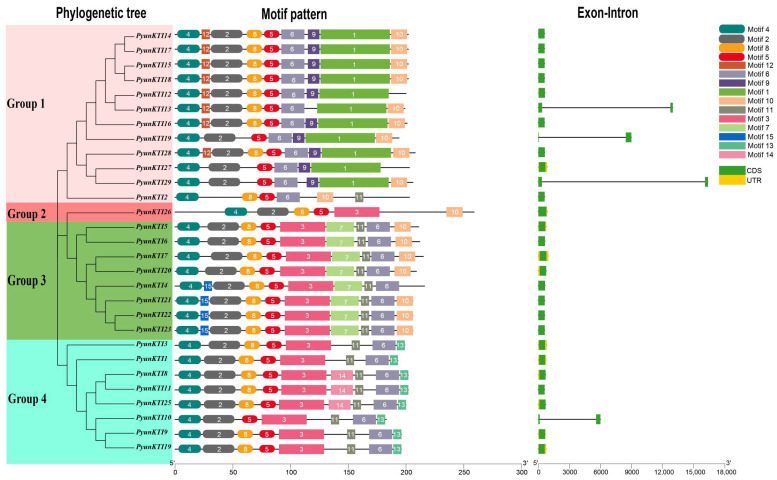
Phylogenetic tree, conserved motif, and gene structure of the PyunKTI proteins. Different colors on the phylogenetic tree represent different groups of Pyun*KTI* genes family. In the motif pattern, the motif numbers 1–15 are displayed in different colored boxes. In exon–intron analysis, the black lines represent introns, the green boxes represent the coding sequences, and the yellow boxes represent the non-coding sequences.

The exon–intron distribution of the 29 *PyunKTI* genes was analyzed (Figure 4). All Kunitz genes do not contain introns, except for *PyunKTI1*, *PyunKTI4*, and *PyunKTI5*, which contain one intron. The analysis of sequence diversity outside of the KTI domain indicates a high degree of conservation within the *KTI* gene family following genome duplication. In total, 15 distinct conserved motifs were identified among the 29 PyunKTI proteins (Figure 4 and Table S7). The number of motifs varied across the PyunKTI proteins, ranging from 6 to 10. However, when considering the phylogenetic tree, it was observed that PyunKTI proteins within the same branch displayed a high similarity in motif distribution. Notably, the group 2 proteins exhibited the fewest motifs (6 motifs), while the group 3 proteins displayed the highest number of motifs (9 or 10 motifs). Conserved motifs 4 and 5 were present in all 29 PyunKTI proteins, while motifs 2, 6, and 8 were consistently found across the PyunKTI proteins. Additionally, certain motifs showed group-specificity: motifs 1, 9, and 12 were unique to nearly all group 1 PyunKTIs, motif 15 was exclusive to the group 3 PyunKTIs, and motif 13 was specific to all group 4 PyunKTIs, with these six motifs being absent in other groups. Based on these results, motifs 2, 4, 5, 6, and 8 were relatively conserved in the *KTI* gene family during evolution.

### 2.4. Chromosomal Location and Duplication of the PyunKTI Gene Family

**Figure 5:**
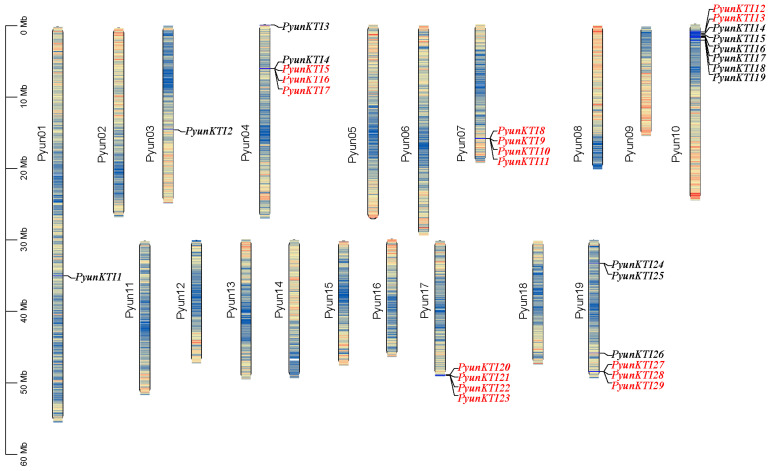
Chromosomal distribution of *P. yunnanensis PyunKTI* genes. Tandem replication genes are shown in red.

The distribution of 29 *PyunKTIs* on the 19 *P. yunnanensis* chromosomes was uneven (Figure 5). There was no correlation between the chromosome length and the number of *PyunKTI* genes. For example, *P. yunnanensis* chromosome 10 was not the longest chromosome but contained eight *PyunKTIs* (*PyunKTI10*-*PyunKTI18*). However, the longest *P. yunnanensis* chromosome 1, contained only one *PyunKTI* (*PyunKTI1*). Detailed information on the chromosomal distribution is shown in Table 1.

As previously noted in the literature, gene duplication frequently accompanies the expansion of gene families [18]. Segmental or whole-genome duplication (WGD) and tandem duplication have been identified as the primary modes of duplication [19]. To gain insight into the mechanisms underlying the expansion of the *KTI*s gene family, we conducted analyses on the chromosomal information from *P. yunnanensis*, *P. trichocarpa*, *A. thaliana*, and *V. vinifera* to examine segmental or WGD duplication, as well as dispersed, proximal, and tandem events. Regarding *PyunKTI*s, 5 segmental or WGD (17.24%), 7 proximal (24.14%), 3 dispersed (10.34%), and 14 tandem duplications (48.28%) genes were detected. In 30 *KTI* genes of *P. trichocarpa*, 16 tandem (55.17%), 6 segmental or WGD (20.69%), 3 dispersed (10.34%), and 4 proximal duplication (13.79%) genes were detected. In seven *Kunitz* genes of *A. thaliana*, two dispersed (28.57%), one proximal (14.29%), and four tandem duplication (57.14%) genes were detected. In five *Kunitz* genes of *V. vinifera*, three tandem (60.00%), one dispersed (20.00%), as well as one proximal (20.00%) duplication gene were detected (Figure S2 and Table S8).

### 2.5. Tandem Repeat and Synteny Gene Pairs

A total of 16 tandem repeat gene pairs were analyzed among the 29 *PyunKTIs* gene members in *P. yunnanensis*. The Ka/Ks ratios of the *KTI* tandem repeat gene pairs ranged from 0.35 to 1.18, with 81.82% of the pairs having Ka/Ks ratios of <1, but the *PyunKTI12*/*PyunKTI13* and *PyunKTI28*/*PyunKTI29* gene pairs had Ka/Ks ratios of >1 (Table S9).

**Figure 6:**
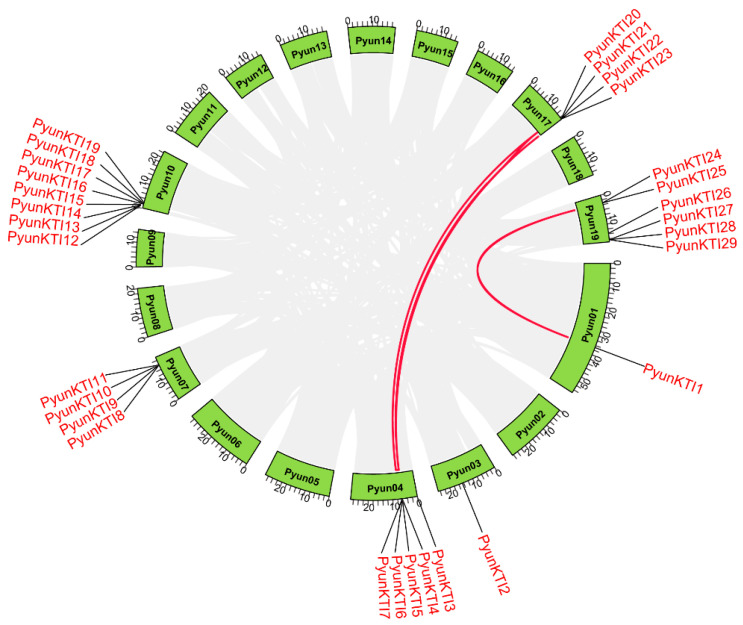
Synteny analysis of the *PyunKTI* genes in *P. yunnanensis*.

**Figure 7:**
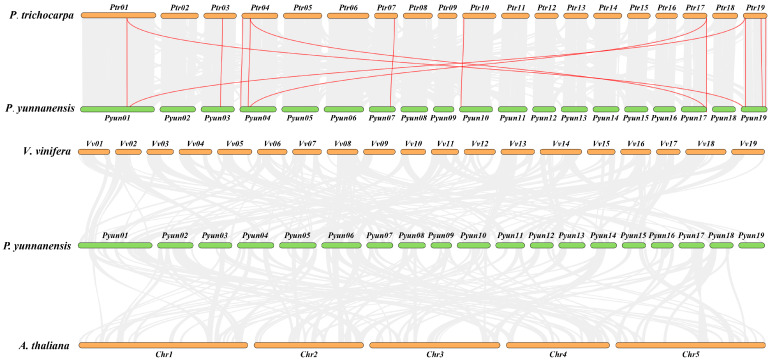
Synteny analysis of the *PyunKTI* genes in *P. yunnanensis*, *P. trichocarpa*, *A. thaliana,* and *V. vinifera*. All collinear genes were labeled gray, while the collinear *KTI* gene pairs were labeled in red.

The synteny of the *KTI* gene family in *P. yunnanensis*, *P. trichocarpa, A. thaliana,* and *V. vinifera* was analyzed. A total of three segmental duplication gene pairs in 29 *PyunKTIs* were found, such as *PyunKTI1/PyunKTI24*, *PyunKTI4/PyunKTI21*, and *PyunKTI5/PyunKTI20*, respectively (Figure 6). There were 21 Synteny *KTI* gene pairs between *P. yunnanensis* and *P. trichocarpa*, 0 pairs between *P. yunnanensis* and *V. vinifera*, and 0 pairs between *P. yunnanensis* and *A. thaliana* (Figure 7). The analysis of the Ka/Ks ratio in the segmental duplication gene pairs of *KTI* revealed a range from 0.15 to 2.79. Notably, 95.65% of the gene pairs exhibited a Ka/Ks ratio below 1, indicating a prevalent occurrence of purifying selective pressure within the *KTI* gene family during evolution. However, it is worth mentioning that the *PyunKTI9/Potri.007G111600.1* gene pair displayed a Ka/Ks ratio greater than 1 (Table S9), suggesting the possibility of positive selection acting on this particular gene pair.

### 2.6. Promoter Analysis of the PyunKTIs Genes

**Figure 8:**
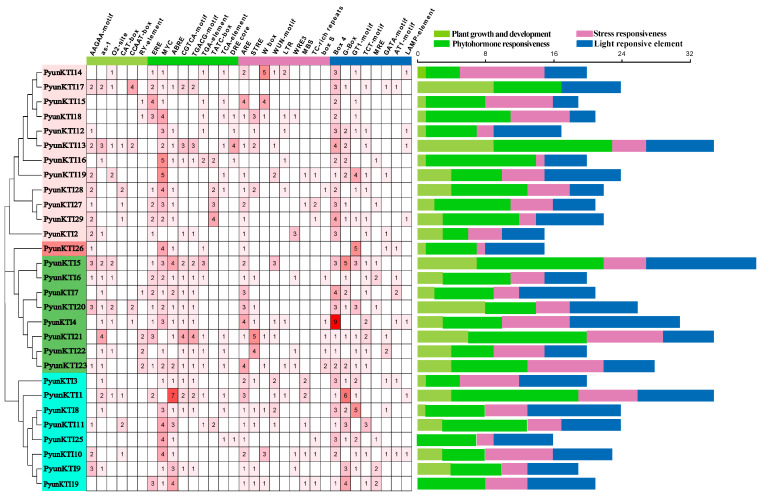
Cis-regulatory element (CRE) analysis of *PyunKTI* genes family. The number of CREs in the promoter region of the *PyunKTI* genes. The number of each CRE was shown in the heatmap box, and white represents that there was no corresponding CRE. Different colors on the bar chart represent different types of CREs in the *PyunKTI* genes family.

The 2000-bp promoters of the 29 *PyunKTI* genes were found to contain four types of cis-acting regulatory elements (CREs) associated with light responsiveness, plant growth and development, phytohormone responsiveness, and stress responsiveness. (Figure 8 and Figure S3 and Table S10). In terms of plant growth and development, it was observed that the promoters of 6 out of the 29 *PyunKTI* genes (20.69%) contained an RY element, which is known for its involvement in seed-specific regulation. Promoters of 16 (55.17%) *PyunKTI* genes may have been involved in secondary xylem development. The MYC binding site involved in drought-inducibility existed in the promoters of 89.66% of *PyunKTI* genes. In addition, the promoters of 17 (58.62%), 22 (75.86%), 20 (68.97%), 16 (55.17%), 13 (44.83%), 5 (17.24%), 12 (41.38%), and 5 (17.24%) *PyunKTI* genes contained ethylene responsiveness (ERE), abscisic acid responsiveness (ABRE), MeJA responsiveness (CGTCA-motif and TGACG-motif), auxin responsiveness (TGA-element), gibberellin responsiveness (TATC-box), salicylic acid responsiveness (TCA-element), and a dehydration responsive element (DRE core), respectively. Furthermore, stress-responsive anoxic-induction elements (ARE), stress-responsive elements (STRE), Fungal elicitor-responsive elements (W box), low-temperature-responsive elements (LTR), MYB binding sites involved in drought-inducibility (MBSs), and defense and stress response elements (TC-rich repeats) were found in the promoters of 25 (86.21%), 14 (48.28%), 8 (27.59%), 8 (27.59%), 7 (24.14%), and 6 (20.69%) *PyunKTI* genes, respectively. Then, three CREs of the stress response regarding the wound-responsive element (WUN-motif, WRE3, box S) were found in the promoters of 10 (34.48%), 7 (24.14%), and 6 (20.69%) *PyunKTI* genes, respectively. The promoters of all 29 *PyunKTI* genes were found to contain light response elements. These findings suggest that the *KTI* genes may play a broad role in responding to diverse stressors and in regulating plant growth and development.

### 2.7. Expression Profile of PyunKTI Genes from P. yunnanensis with Upright and Inverted Cuttings

**Figure 9:**
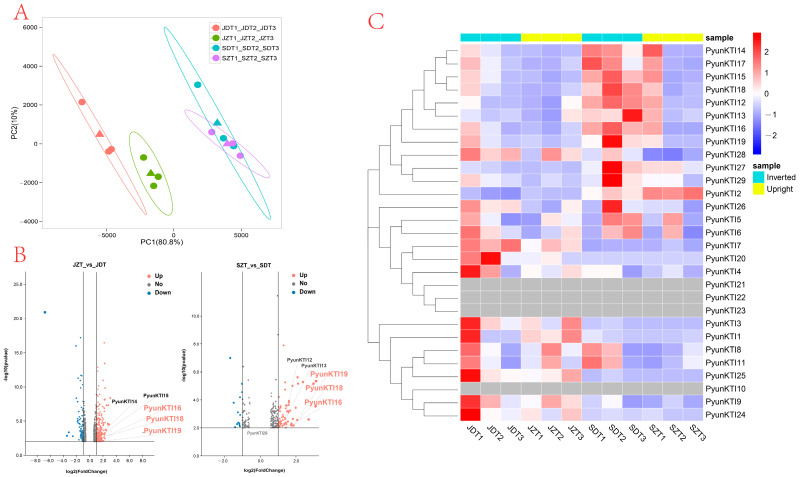
Expression profile of *PyunKTI* genes from *P. yunnanensis* with upright and inverted cuttings. (**A**) PCA analysis of the *PyunKTI* genes in *P. yunnanensis.* (**B**) Volcano plot analysis of the *PyunKTI* genes in *P. yunnanensis*. (**C**) Heatmap analysis showing the expression patterns and coexpressed relationships of each *KTI* gene.

This article sequenced and analyzed the raw transcriptome reads to assess the differences between the primary and fast-growing periods of upright and inverted cuttings. An RNA-seq analysis of the two processed cuttings (Figure 9) revealed that the expression levels in upright and inverted cuttings were markedly different at the same growth period. The top two principal components (PCs) obtained in the principal components analysis (PCA) could explain 90.8% of the total variation (Figure 9A). Notably, principal component 1 (PC 1) accounted for a substantial 80.8% of the observed variation, effectively distinguishing the different growth periods. Furthermore, principal component 2 (PC 2), explaining 10% of the variation, successfully differentiated the leaf samples obtained from the upright and inverted cuttings. These findings demonstrate significant differences in the expression patterns of *PyunKTI* genes across various growth periods while also revealing subtle variations associated with the direction of the cuttings.

As depicted in Figure 9B,C, a considerable number of *PyunKTI* genes exhibited higher expression levels in the inverted cuttings compared to the upright cuttings during the same growth period. Taking group 1 as an example, *PyunKTI16*, *PyunKTI18*, and *PyunKTI19* were found to be upregulated in both upright and inverted cuttings, as evidenced by the gene expression data obtained from the leaves. In the volcano plot, in the comparison of the JZT vs. JDT group and SZT vs. JDT group in July, log2 (FoldChange) > 1 was used as the criteria to screen for differential genes. *PyunKTI16*, *PyunKTI18*, and *PyunKTI19* were all significantly upregulated.

### 2.8. RT-qPCR Analysis of the PyunKTI Genes Expression Level

**Figure 10:**
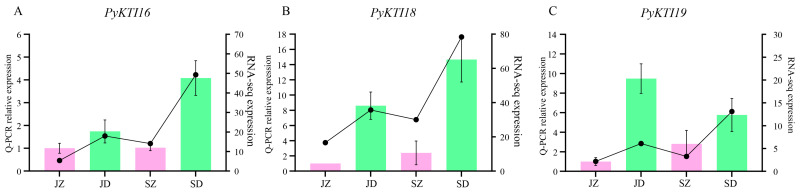
Inverted treatment increases the expression of *KTI* genes. (**A**–**C**) show the qPCR relative expression and transcriptome results of *PyunKTI16*, *PyunKTI18*, and *PyunKTI19*, respectively. JZ, JD, SZ, and SD denote July upright insertion, July inversion insertion, September upright insertion, and September inversion insertion, respectively. Pink represents the upright insertion results, green represents the inversion insertion results, and the broken line represents the transcriptome results.

The expression levels of the *PyunKTI16*, *PyunKTI18*, and *PyunKTI19* genes under upright and inversion insertion treatments are shown in Figure 10. The results showed that, in the case of upright insertion, the expression of genes increased slightly with the change in developmental stage, from the early growth period to the rapid growth period. However, at the same developmental stage, the gene expression in the inversion insertion treatment increased sharply. Consistent results were obtained in the analysis of the transcriptome data, which showed that *PyunKTI16*, *PyunKTI18*, and *PyunKTI19* had significant responses to inverted stress.

### 2.9. Transcriptome Analysis in GEO Database of PyunKTI Genes

**Figure 11:**
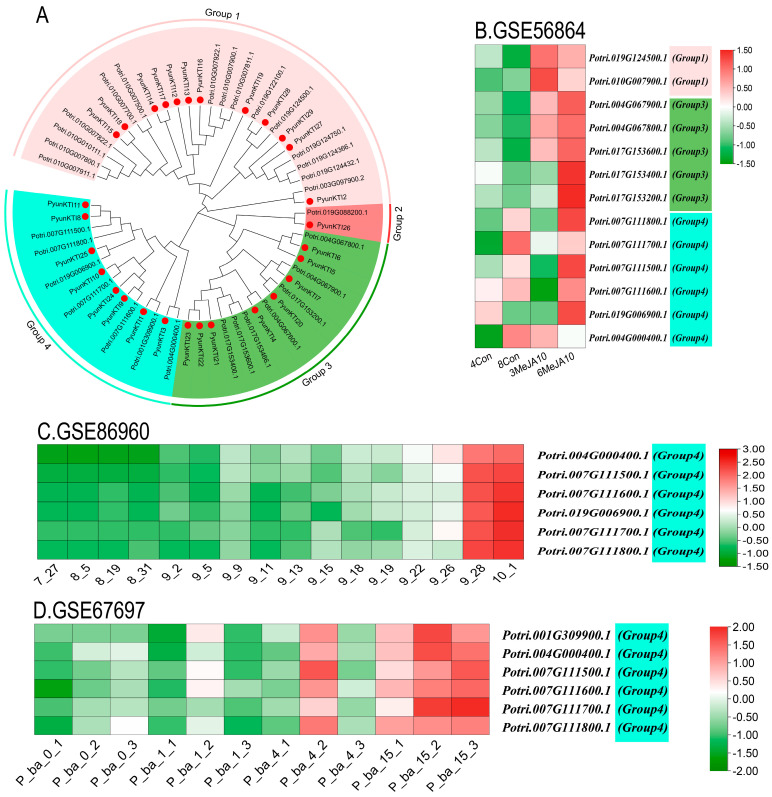
*KTI* gene expression in the GEO database in the related transcriptome with *P. trichocarpa* as the reference gene. (**A**) NJ Phylogenetic tree of *P. yunnanensis* and *P. trichocarpa*. (**B**) GSE56864: Effect of Methyl Jasmonate on the poplar root transcriptome. 4Con and 8Con are the control roots; 3MeJA10 and 6MeJA10 are the Methyl Jasmonate treated roots. (**C**) GSE86960: A time series of autumn senescence leaves from *P. tremula*. Samples were collected from the growing season in July to the aging season in October. (**D**) GSE67697: Transcriptome analysis of poplar during leaf spot infection with *Sphaerulina* spp. Samples were collected at 0, 1, 3, 4, and 15 days after infection with the pathogen.

**Figure 12:**
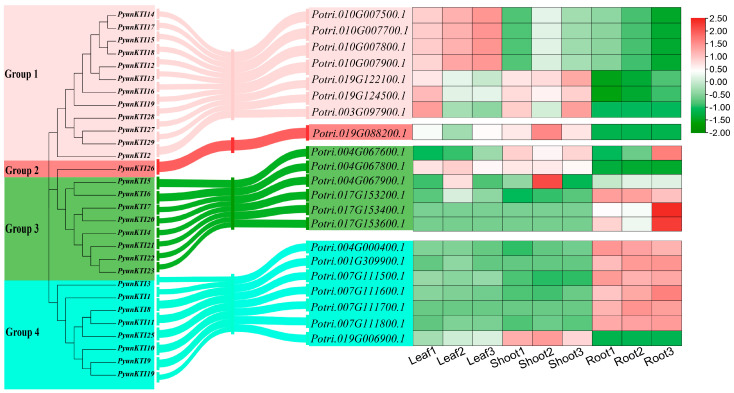
The grouping relationship between the *KTI* gene of *P. yunnanensis* and the *KTI* gene of *P. trichocarpa* and the expression pattern of the *KTI* gene in various tissue parts of *P. trichocarpa*.

The phylogenetic tree on the *KTI* gene family of *P. yunnanensis* and *P. trichocarpa* was grouped based on homologous multiple sequence comparisons (Figure 11A). Different levels of expression in major tissues (shoot tip, leaf, and root) of the *KTI*s in *P. trichocarpa* were analyzed by RNA-seq (GSE81077). The *Kunitz* genes of *P. trichocarpa* were divided into four groups (group 1/2/3/4) in the phylogenetic tree of *P. yunnanensis* and *P. trichocarpa* (Figure 12).

**Figure 13:**
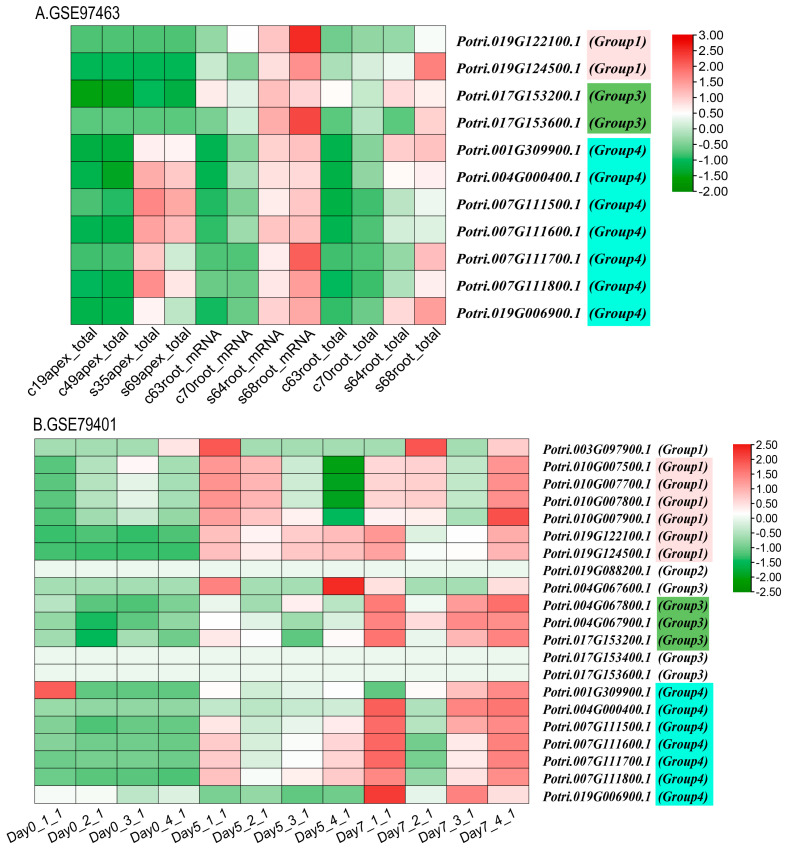
*KTI* gene expression in the transcriptome of drought-treated *P. trichocarpa* as a reference gene in the GEO database. (**A**) GSE97463: Differential gene expression analysis of drought-responsive sense and antisense genes in *Populus*. Sample name starting with c represents the control group, and sample name starting with s represents the drought treatment group. (**B**) GSE79401: RNA-seq of drought-treated *P. trichocarpa*. Samples were collected at 0, 5, and 7 days for the drought treatment.

The functional expression of *KTI* genes in *P. yunnanensis* under different stress treatments was analyzed to screen and classify the *KTI* genes in the transcriptome in *P. trichocarpa*. Groups 1/2/3/4 may play different functions. In the GSE56864 data (Figure 11B), the *PyunKTI* genes were significantly upregulated after the Methyl Jasmonate treatment of sample roots, which were mainly concentrated in groups 3 and 4. In the GSE86960 dataset (Figure 11C), a gene expression analysis was conducted to examine the changes in *P. tremula* during autumn senescence. Members of the *KTI* gene family were gradually upregulated with time, with the highest gene expression at the end of leaf senescence, and these genes were concentrated only in group 4, from late summer and late into autumn. Using the GSE67697 data (Figure 11D), we conducted a transcriptome analysis of poplar during leaf spot infection with *Sphaerulina* spp. After inoculation with *Sphaerulina* spp., members of the *KTI* gene family were progressively upregulated with increasing time, reaching the highest gene expression at 15 days, and these genes were concentrated only in group 4. In the GSE97463 data (Figure 13A), differential gene expression analysis of drought-responsive sense and antisense genes in *Populus* was noted. In the short-term drought treatment, members of the *KTI* gene family were significantly upregulated in the apex, concentrated only in group 4. Regarding the long-term drought treatment, members of the *KTI* gene family were significantly upregulated in the roots, mainly concentrated in group 4, with some members upregulated in group 3 and group 1. Regarding the GSE79401 data (Figure 13B), plants on day 5 were under a mild drought state, and on day 7, they were in a severe drought state. Members of the *KTI* gene family were already significantly upregulated under mild drought, mainly in group 1 and group 4. Under prolonged drought treatment, more members of the *KTI* gene family were upregulated and more highly expressed, mainly in groups 1, 3, and 4.

## 3. Discussion

### 3.1. Conserved Fundamental Features and Diverse Expression Patterns of KTIs

The traditional classes of plant protein protease inhibitors encompass various types, such as Kunitz, Bowman–Birk, potato I and II, squash, barley, cystatins, and miscellaneous. Since the discovery of soybean *KTI* in 1945, numerous other inhibitors have been identified and classified as *KTIs* due to their characteristic structural features. These features include a molecular weight ranging from approximately 18 to 24 kDa, two disulfide bonds, a single reactive site, multiple β-trefoil folds, and most importantly, the presence of the trypsin inhibitor domain [20,21]. In this study, we found that *PyunKTI2* proteins of *P. yunnanensis* exhibited high sequence similarities to other known *KTIs* [11,22,23] in conserved regions, which were recognized as structural features of *KTIs*.

The number of *KTI* family members and the level of activity exhibited significantly. The number of *KTI* family members in the *Populus* species is more than in other species and the *KTI* members found in *A. thaliana* and *V. vinifera* were fewer than 25% in *P. yunnanensis*. Most of the *KTI* genes in the 16 species existed as tandem repeats, and the genes underwent purifying selection within *P. yunnanensis* and between *P. yunnanensis* and *P. trichocarpa*. Gene family expansion is often associated with gene duplication, which occurs through two main patterns: WGD and tandem duplication [18]. In this study, the expansion of the *P. yunnanensis*, *P. trichocarpa*, *A. thaliana,* and *V. vinifera KTI* gene families occurred mainly through tandem repeat events. The chromosomal localization results showed that 29 *KTI*s were unevenly distributed on seven chromosomes (Chr 1, Chr 3, Chr 4, Chr 7, Chr 10, Chr 17, and Chr 19). *KTI1, KTI2,* and *KTI3* were present on chromosomes individually, while all the other genes were present in clusters of genes, which reflects the selection of *KTI*s during gene amplification.

The statistical results of ZHANG et al. [24] showed that most angiosperms contain *KTI* members, and the number of *KTI* members of monocotyledon and dicotyledon plants is significantly different. The number of *KTI* members of monocotyledon plants is generally small, only 1 to 3, while the number of *KTI* members of dicotyledon plants is more; for example, *Medicago sativa* has the largest number of *KTI* members (51), followed by *Glycine max* (50). In this paper, we analyzed the *KTI* gene members of 13 species of *Populus and Salix*, which belong to the most abundant type of dicotyledonous plants. It is speculated that *KTI* plays a more important role in the growth and development of dicotyledonous plants, especially Salicaceae.

An analysis of the conserved structural domains revealed that the number and type of conserved motifs varied among the *PyKTI*s. However, gene members within the same group shared similar conserved motif types, suggesting the presence of analogous structures and functions within the same group [25]. All *PyKTI*s contained motifs 4, 2, and 5, which may represent the key elements conserved during evolution. Motif 7 is exclusive to group 3, while motifs 1, 9, and 12 are found only in group 1, and motifs 13 and 14 are also unique to group 1. The similar motif composition within the same group suggests that the proteins are structurally conserved and likely share similar functions.

The overall secondary structure of the Kunitz inhibitor family consists of 43% β structures, 55% loops, and 2% helices, and more than 50% of the structural part is made up of loops [20]. This could be the reason behind the dynamic nature of these proteins. The average secondary structure of the 29 *PyunKTI* proteins was composed of 11.17% α-helix, 32.63% extended strand, 6.83% beta turn, and 49.37% random coil. The secondary structure of the poplar *KTI* is different from other types of *KTI* genes. The α-helix structure is composed of a segment of MK***FLL*A (M: Methionine; K: Lysine; F: Phenylalanine; L: Leucine; A: Alanine) at the front end of the gene, and the α-helix protein is the main part of the transmembrane protein. The alpha-helical structure of a protein present in the cellular lipid bilayer, as a transmembrane protein, usually has both hydrophobic and hydrophilic side chains. The hydrophobic side chains interact with the lipids present in the membrane, while the hydrophilic side chains remain exposed to the outer surface.

As a subset of the serine protease inhibitors, Kunitz participates in the regulation of many stress-resistant processes, such as plant growth and development, the synthesis of secondary metabolites, and plant hormone signaling pathways and their interactions [20]. In this study, the characteristics of the *KTI* family in *P. yunnanensis* and the regulatory role of *KTI* in response to the inverted cuttings’ stress were analyzed, providing gene resources for *P. yunnanensis* resistance breeding and advancing the functional study of the *P. yunnanensis KTI* genes.

### 3.2. Involved in Plant Growth and Development

Because plant *KTIs* can regulate the activity of serine protease, avoid excessive protein degradation, and promote cell homeostasis, it has received more and more attention in recent years, and many reports indicate that it is closely related to plant growth and development. It has been found that serine protease plays an important role in plant stress, senescence, programmed cell death, and sexual reproduction [26]. *Phaseolus vulgaris* subtilisin-like protease 2 (PvSLP2) was much more active in aged leaves than in mature and young leaves, which was related to the degree of leaf senescence [27]. Islam et al. found that the decreased expression of Trifolium repens Kunitz proteinase inhibitor 2 (*Tr-KTI2*) affected a series of developmental traits, including the stem length, branch number, and petiole length, as well as increased morphological diversity and decreased developmental stability, indicating that *KTI* plays an important role in genetic variation and the developmental stability of plants [28]. Ethylene and mechanical injury can activate the transcriptional activity of HECATE 1 (HEC1), a member of the *A. thaliana* bHLH transcription factor family, and promote Kunitz protease inhibitor 1 (Kunitz-PI1) accumulation of apical curls and cotyledon apices [29,30]. In this study, transcriptome and qRT-PCR at primary and rapid growth showed that *PyunKTI1*, *PyunKTI2,* and *PyunKTI4* were upregulated during the growth process of *P. yunnanensis.*

### 3.3. Involvement in Biological and Environmental Stresses

*KTI* can also enhance plants’ tolerance to various stresses, including drought, salinity, and low temperatures [28,31]. We hypothesized that some *PyKTI* genes might be regulated by stress because their promoters contain cis-acting elements related to stress responses. For example, we found that the promoters of *PyKTI3* and *PyKTI1* contain two MBSs, and the promoters of *PyKTI11, PyKTI25*, and *PyKTI10* contain four MYCs. All five *PyKTIs* belong to group 4 in the evolutionary tree, and MBSs and MYCs are cis-acting elements related to drought stress (Figure 8). *Potri.001G309900.1*, *Potri.004G000400.1*, *Potri.007G111500.1*, *Potri.007G111600.1*, *Potri.007G111700.1*, *Potri.007G111800.1*, and *Potri.019G006900.1* in *P. trichocarpa* also belonged to group 4 in the evolutionary tree, and these seven genes showed upregulated expression in both the apex and roots of *P. trichocarpa* after the drought treatment and continued to be upregulated as the drought treatment continued (Figure 11A and Figure 13).

Numerous studies have shown that conditions like high temperatures, water stress, and the application of Methyl Jasmonate significantly elevate the mRNA and protein levels of *KTI* in various plants, including *Brassica oleracea*, *Glycine max*, *Solanum tuberosum*, and *Passiflora edulis*. These findings indicate that *KTI* plays a crucial role in the response to abiotic stress [12,32,33,34,35]. *PtKTI12* is important for wobble uridine modification and stress tolerance under water deficit conditions [36]. We analyzed the expression of *Cassia obtusifolia CoTI* under different stress conditions and found that its expression was significantly affected by salt stress, drought, and abscisic acid [37]. Islam et al. used RNA interference technology to reduce the mRNA content of the *KTI* gene in *Trifolium repens*, which resulted in the destruction of homeostasis and the release of a large number of intracellular emergency molecules (H_2_O_2_) and activated the flavonoid metabolic pathway [28]. Sustained expression of the trypsin protease inhibitor in transgenic tobacco confers greater resistance to salt stress, acid-base stress, and drought stress [31]. In this paper, the inversion of *P. yunnanensis* is a type of stress compared with plants, and its survival mechanism has not been clearly studied. However, transcriptome and qRT-PCR studies of upright cuttings and inverted cuttings showed that the upregulation response of *KTI* family members was obvious in inverted cuttings. Accordingly, we hypothesized that the *KTI* genes exert the same stress tolerance function in *P. yunnanensis*.

## 4. Materials and Methods

### 4.1. Identification and Characterization of the PyunKTI Gene Family

*Arabidopsis thaliana* and *Vitis vinifera* were selected as model species. The *A. thaliana*, *V. vinifera*, *Salix*, and *Populus* genomes were downloaded from Phytozome, NCBI, and PlantGe-nIE. A hidden Markov model (HMM) was used to identify the Kunitz candidates, and the HMM profiles of the Kunitz (PF00017) domains were downloaded from the Pfam protein database (https://www.ebi.ac.uk/interpro/, accessed on 15 June 2024) [38]. The putative Kunitz proteins, identified by HMMER v3.0 software [39], were submitted to the CDD database and BLAST program (https://www.ncbi.nlm.nih.gov/Structure/cdd/wrpsb.cgi, accessed on 15 June 2024) to check for complete Kunitz domains [40]. The family members of *P. yunnanensis* were confirmed and sorted according to their chromosomal position for downstream analysis. The physicochemical properties of the putative Kunitzs, including the number of amino acids (No. of aa), molecular weight (MW), isoelectric point (pI), instability index, and grand average of hydropathicity (GRAVY), were analyzed using the online ExPASy-ProtParam tool (http://web.expasy.org/protParam/, accessed on 15 June 2024). The subcellular localization, secondary structure, and signal peptides of PyunKTI proteins were predicted using Plant-mPloc, Prabi, and SignalP 6.0, respectively. Homologous proteins in the PDB database were downloaded using the PDB file, using the ESPript3.0 beautify protein ratio on file, and adding a secondary structure.

### 4.2. Analyses of Phylogenetics, Gene Structure, Motif Composition, Chromosomal Distribution, Gene Duplication, and Synteny

To investigate the evolutionary relationships among the 301 candidate Kunitz proteins from 13 plant species, multiple sequence alignments were conducted using ClustaW with default parameters [41]. Subsequently, a phylogenetic tree was constructed using MEGA 11 software, employing the neighbor-joining (NJ) method with pair-wise deletion, Poisson correction, and 1000 bootstrap replicates for statistical support [42,43]. The resulting phylogenetic tree was visualized using the EvolView v3 software [44]. Furthermore, comprehensive analyses were performed to explore additional aspects of the Kunitz proteins, including the gene structure, motif composition, chromosomal distribution, gene duplication, and synteny.

The genomic DNA sequences and coding sequences (CDS) of the 29 *PyunKTI* genes were utilized to investigate the gene structure in *P. yunnanensis*, a process that involves analyzing the arrangement of various subunits within the gene. To identify and characterize the conserved motifs in the Kunitz proteins, we employed the online Multiple Expectation Maximization for Motif Elicitation tool (MEME). The MEME tool uses statistical algorithms and machine learning techniques to identify patterns or motifs within protein sequences. In this study, we set the parameters to search for 15 motifs with the optimum motif width of 8–80 residues, allowing for any number of repetitions [45]. Tattoos were used to visualize the gene structure and motif composition of PyunKTI proteins [46]. The chromosomal positions of the *PyunKTIs* were mapped using MapChart v5.6.0 software, and the Multiple Collinearity Scan toolkit (MCScanX) was used to analyze the duplication pattern and synteny of the *PyunKTIs* [47]. Finally, to assess the selective pressure acting on the *PyunKTIs*, we calculated the ratio of nonsynonymous to synonymous substitutions (Ka/Ks) using KaKs_Calculator2.0 [48].

### 4.3. Identification of Cis-Regulatory Elements (CREs) in PyunKTIs Gene Promoters

To identify cis-regulatory elements (CREs) in the promoter regions of the 29 *PyunKTI* genes, the 2000-bp sequences upstream from the start codon were analyzed using the PlantCARE tool [49]. The obtained results were further visualized using EvolView v3 [44].

### 4.4. Transcriptome Analysis About PyunKTI Genes

In the Gene Expression Omnibus (GEO) database, transcriptome items were screened for “poplar” keywords and various stress treatments. These GEO data are all based on *P. trichocarpa* as the reference genome, and *P. trichocarpa* and *P. yunnanensis* were constructed as a phylogenetic tree and classified according to the previous groups. The expression datasets of the Kunitz gene family in different treatments were constructed. GSE81077 provides data for the RNA-seq of major tissues and xylem cell types of *P. trichocarpa* [50]. GSE56864 focuses on the effect of Methyl Jasmonate on the poplar root transcriptome [51]. GSE86960 analyzes the expression of a gene in *P. tremula* in senescent leaves from different years [52]. GSE67697 investigates the transcriptome analysis of poplar during leaf spot infection with *Sphaerulina* [53]. GSE97463 explores the differential gene expression analysis of drought-responsive sense and antisense genes in *Populus* [54]. GSE79401 provides RNA-seq data for drought-treated *P. trichocarpa* [55]. Xylem tissue was collected from the plants (1) without drought treatment (control, day 0), (2) under mild drought stress (stage 3, day 5), (3) under severe drought stress (stage 4, day 7), and was followed by RNA-seq analysis.

### 4.5. The Expression Profiles of PyunKTI Genes Were Analyzed Using the Available Transcriptome Datasets

The cuttings of 3 clones of *P. yunnanensis* were cultivated in the greenhouse for one year (Southwest Forestry University, Kunming, China. 102.76 E, 25.06 N). In February 2022, we obtained about 15 cm cuttings and preserved these in the soil. The cuttings with different tissues were used in the experiments performed in July 2023 (the early growth period). We obtained morphologically uniformly sized roots, as well as stems, lateral buds, branches, young leaves (first to third leaves counted from the stem tip), and stem tips. All plant samples collected were brought back in liquid nitrogen and then stored at −80 °C until their next use.

One clone was treated as a biological replicate, and three replicates were established. All samples were sent to BioMark Technologies (Beijing, China) for RNA-seq. The genome of *P. yunnanensis* was used as a reference annotation library for data analysis (BioProject: PRJNA886471). Fragments per kilobase of transcript per million mapped (FPKM) were used as an index to measure the expression level of the transcripts or genes. Based on the transcriptome datasets, the expression level of the *Kunitz* genes in differential expression levels of genes under different growth stages of *P. yunnanensis* between positive and inverse insertions was converted with log2 (FPKM).

### 4.6. RT-qPCR Analysis of the PyunKTI Genes

Gene-specific primers for *PyuKTI16, PyuKTI18*, and *PyuKTI19* were obtained from the study using Primer-BLAST [56], and the primer details can be found in Table S11, which provide information regarding the primers used for amplification. To investigate the dynamic expression patterns of the three *KTI* genes, reverse-transcription quantitative PCR (RT-qPCR) was performed. For normalization purposes, we chose the endogenous control gene HIS as the internal standard. The Ct values were amplified and quantified using the Rotor-Gene qPCR system, utilizing the Fast Super EvaGreen qPCR Master Mix (US Everbright Inc., Suzhou, China). Two-step amplification was carried out as follows: 95 °C for 2 min, followed by 40 cycles of 95 °C for 10 s, and 60 °C for 30 s. This experiment was repeated for three biological replicates, each involving three technical repeats. To determine the relative expression levels, the 2^−ΔΔCt^ method was employed [57].

## 5. Conclusions

We identified 29 *PyunKTI* genes from *P. yunnanensis*, which can be classified into four groups. Tandem repeat events mainly achieve the amplification of *PyunKTI* genes. Biologically, *PyunKTI* plays a wide range of roles in coping with various stresses and regulating plant growth and development. The results of this study will contribute to a better understanding of the functional and molecular mechanisms of the *PyunKTI* genes in stress resistance and their potential use in the genetic evolution of *P.yunnanensis*.

## Figures and Tables

**Table 1:** Identification and characterization of the 29 PyunKTIs proteins.

Gene Name	Gene ID	Genomics Position	Amino AcidsNumber(aa)	Molecular Formula	Isoelectric Point	Molecular Weight	Instability Index	Aliphatic Index	GRAVY	Subcellular Localization
*PyunKTI1*	*Pyun01G029930*	Chr01: 34845724-34846485	193	C_933_H_1468_N_248_O_273_S_11_	8.18	20,880.08	61.68	89.95	0.25	Vacuole.
*PyunKTI2*	*Pyun03G009060*	Chr03: 14540624-14541235	203	C_1020_H_1588_N_262_O_295_S_12_	5.33	22,626.13	32.53	98.87	0.16	Vacuole.
*PyunKTI3*	*Pyun04G000040*	Chr04: 55635-56439	199	C_958_H_1482_N_252_O_299_S_9_	4.41	21,602.35	46.52	81.36	−0.05	Vacuole.
*PyunKTI4*	*Pyun04G005750*	Chr04: 6150656-6151306	216	C_1075_H_1670_N_278_O_307_S_12_	6.43	23,785.48	41.02	84.86	−0.07	Vacuole.
*PyunKTI5*	*Pyun04G005770*	Chr04: 6159791-6160553	211	C_1058_H_1648_N_262_O_315_S_7_	5.09	23,302.71	42.68	98.34	0.08	Vacuole.
*PyunKTI6*	*Pyun04G005780*	Chr04: 6172938-6173586	212	C_1057_H_1643_N_261_O_315_S_7_	4.96	23,271.65	42.54	96.93	0.11	Vacuole.
*PyunKTI7*	*Pyun04G005790*	Chr04: 6182198-6183153	215	C_1039_H_1659_N_271_O_318_S_8_	5.48	23,291.71	35.00	95.58	0.09	Vacuole.
*PyunKTI8*	*Pyun07G011750*	Chr07: 15976464-15977166	202	C_1006_H_1578_N_276_O_282_S_9_	8.27	22,339.82	43.52	95.50	0.13	Vacuole.
*PyunKTI9*	*Pyun07G011760*	Chr07: 15991434-15992125	196	C_920_H_1436_N_246_O_278_S_10_	4.58	20,711.60	40.41	83.06	0.09	Vacuole.
*PyunKTI10*	*Pyun07G011770*	Chr07: 15993379-15999370	183	C_879_H_1380_N_246_O_268_S_9_	5.41	19,970.65	48.54	79.34	−0.09	Vacuole.
*PyunKTI11*	*Pyun07G011780*	Chr07: 16001371-16001979	202	C_1004_H_1573_N_275_O_284_S_9_	7.58	22,328.75	41.01	93.56	0.11	Vacuole.
*PyunKTI12*	*Pyun10G000910*	Chr10: 1058936-1059577	200	C_975_H_1523_N_247_O_291_S_10_	5.15	21,681.90	30.23	89.65	0.23	Vacuole.
*PyunKTI13*	*Pyun10G000920*	Chr10: 1070920-1083898	199	C_969_H_1504_N_244_O_291_S_10_	4.86	21,548.66	36.54	90.60	0.30	Vacuole.
*PyunKTI14*	*Pyun10G000940*	Chr10: 1273093-1273701	202	C_975_H_1525_N_245_O_287_S_11_	4.94	21,623.96	26.31	93.07	0.35	Vacuole.
*PyunKTI15*	*Pyun10G000990*	Chr10: 1485338-1485946	202	C_980_H_1532_N_242_O_293_S_12_	4.72	21,777.11	27.63	93.07	0.30	Vacuole.
*PyunKTI16*	*Pyun10G001030*	Chr10: 1649193-1649798	201	C_982_H_1523_N_241_O_296_S_12_	4.79	21,826.05	38.14	86.32	0.17	Vacuole.
*PyunKTI17*	*Pyun10G001050*	Chr10: 1730499-1731107	202	C_968_H_1518_N_246_O_289_S_11_	4.94	21,578.84	28.91	91.63	0.30	Vacuole.
*PyunKTI18*	*Pyun10G001070*	Chr10: 1821744-1822352	202	C_977_H_1528_N_242_O_291_S_12_	4.81	21,705.05	26.67	93.07	0.31	Vacuole.
*PyunKTI19*	*Pyun10G001220*	Chr10: 2244406-2253386	194	C_946_H_1458_N_242_O_287_S_9_	5.00	21,101.97	30.27	81.39	0.06	Vacuole.
*PyunKTI20*	*Pyun17G015360*	Chr17: 18745402-18746164	209	C_1033_H_1660_N_268_O_295_S_11_	9.17	22,906.82	36.39	95.60	0.06	Vacuole.
*PyunKTI21*	*Pyun17G015370*	Chr17: 18797532-18798152	206	C_1027_H_1641_N_283_O_285_S_10_	9.59	22,833.65	27.43	88.01	−0.10	Vacuole.
*PyunKTI22*	*Pyun17G015380*	Chr17: 18822863-18823483	206	C_1032_H_1647_N_281_O_281_S_10_	9.67	22,807.74	29.10	90.39	0.00	Vacuole.
*PyunKTI23*	*Pyun17G015390*	Chr17: 18827204-18827824	206	C_1032_H_1647_N_281_O_281_S_10_	9.67	22,807.74	29.10	90.39	0.00	Vacuole.
*PyunKTI24*	*Pyun19G002480*	Chr19: 3226565-3227324	196	C_931_H_1460_N_248_O_279_S_10_	4.60	20,911.93	48.91	88.52	0.15	Vacuole.
*PyunKTI25*	*Pyun19G002500*	Chr19: 3248588-3249293	200	C_979_H_1519_N_267_O_283_S_11_	5.53	21,910.11	57.69	83.80	0.01	Vacuole.
*PyunKTI26*	*Pyun19G010740*	Chr19: 15803407-15804259	259	C_1262_H_1993_N_309_O_384_S_9_	4.71	27,927.09	32.92	107.92	0.33	Vacuole.
*PyunKTI27*	*Pyun19G013290*	Chr19: 18393576-18394424	203	C_989_H_1543_N_253_O_292_S_13_	4.99	22,066.43	39.30	90.69	0.31	Vacuole.
*PyunKTI28*	*Pyun19G013300*	Chr19: 18397269-18397895	208	C_984_H_1537_N_253_O_310_S_14_	4.54	22,320.38	33.11	83.37	0.18	Vacuole.
*PyunKTI29*	*Pyun19G013310*	Chr19: 18402299-18418665	206	C_987_H_1539_N_251_O_299_S_16_	4.64	22,218.54	35.10	84.61	0.29	Vacuole.

## Data Availability

Genomic data and RNA-seq data for *P. yunnanensis* are available from the corresponding author. The data supporting the results of this study are available from the corresponding author upon reasonable request.

## Supplementary Materials

The following supporting information can be downloaded at: https://www.mdpi.com/article/10.3390/ijms26010188/s1.
