# Supplementary material for: Identification of Kunitz-Type Inhibitor Gene Family of Populus yunnanensis Reveals a Stress Tolerance Function in Inverted Cuttings
Source: Int J Mol Sci. 2024 Dec 29;26(1):188. doi: 10.3390/ijms26010188 (PMC11720115; doi:10.3390/ijms26010188)
Supplement: Supplementary file 1 [file ijms-26-00188-s001.zip › Figure S1.pdf]

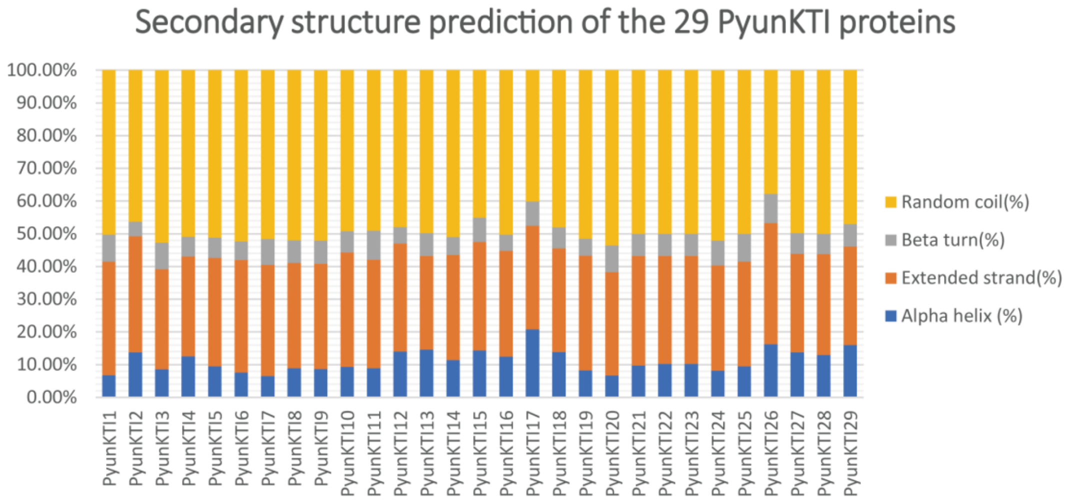

Figure S1. The secondary structure of the 29 PyunKTI proteins were composed an α-helix, Extended strand, Beta turn and Random coil.
