# Supplementary material for: Identification of Kunitz-Type Inhibitor Gene Family of Populus yunnanensis Reveals a Stress Tolerance Function in Inverted Cuttings
Source: Int J Mol Sci. 2024 Dec 29;26(1):188. doi: 10.3390/ijms26010188 (PMC11720115; doi:10.3390/ijms26010188)
Supplement: Supplementary file 1 [file ijms-26-00188-s001.zip › Figure S2.pdf]

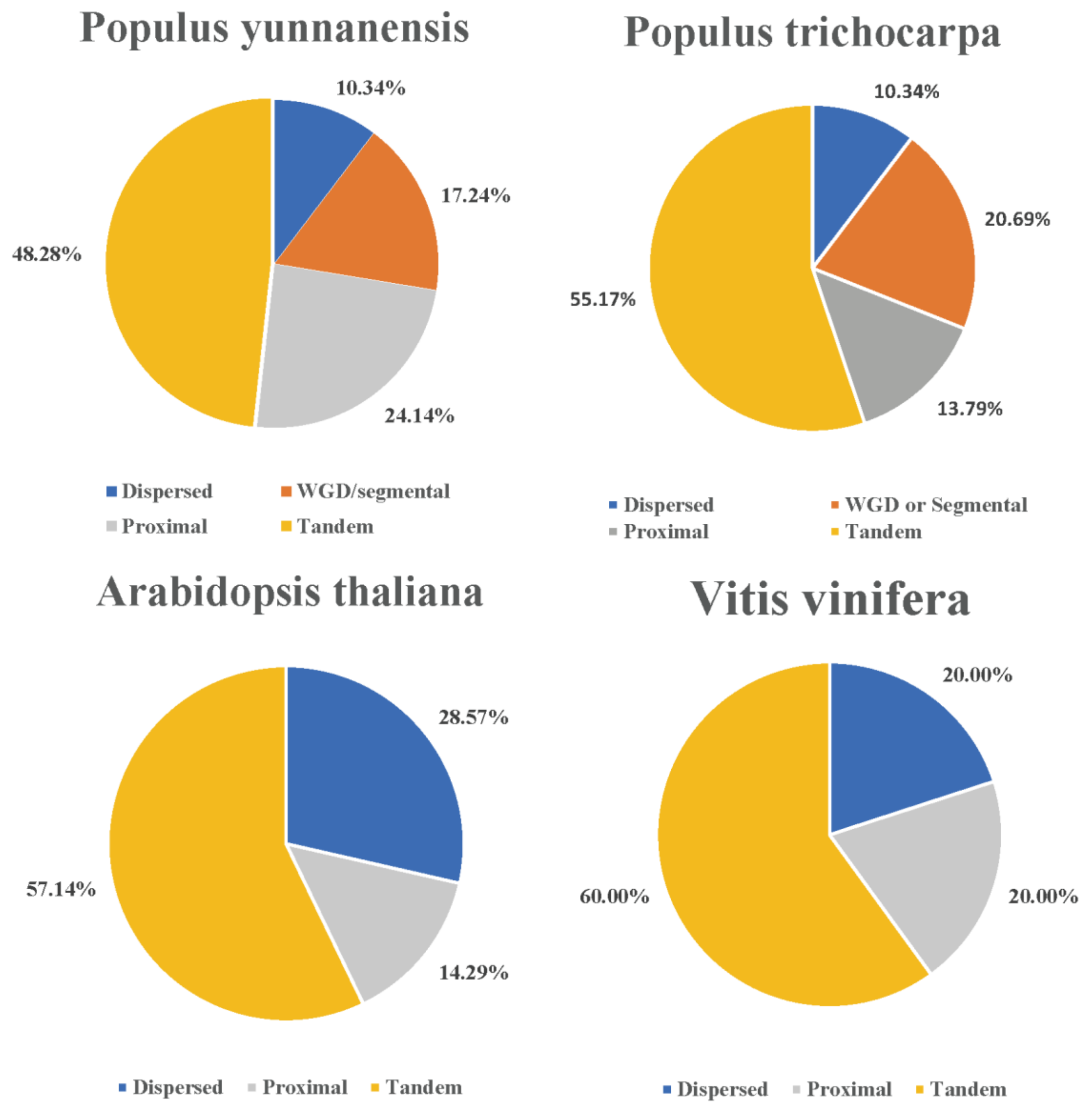

Figure S2. Expansion mechanisms of the Kunitz gene family in *Populus yunnanensis, Populus trichocarpa, Arabidopsis thaliana* and *Vitis vinifera*. Different colors in the pie chart correspond to different types of gene duplication. WGD or segmental: segmental duplication/whole-genome duplication; dispersed: dispersed duplication; proximal: proximal duplication; tandem: tandem duplication.
