# Supplementary material for: Identification of Kunitz-Type Inhibitor Gene Family of Populus yunnanensis Reveals a Stress Tolerance Function in Inverted Cuttings
Source: Int J Mol Sci. 2024 Dec 29;26(1):188. doi: 10.3390/ijms26010188 (PMC11720115; doi:10.3390/ijms26010188)
Supplement: Supplementary file 1 [file ijms-26-00188-s001.zip › Figure S3.pdf]

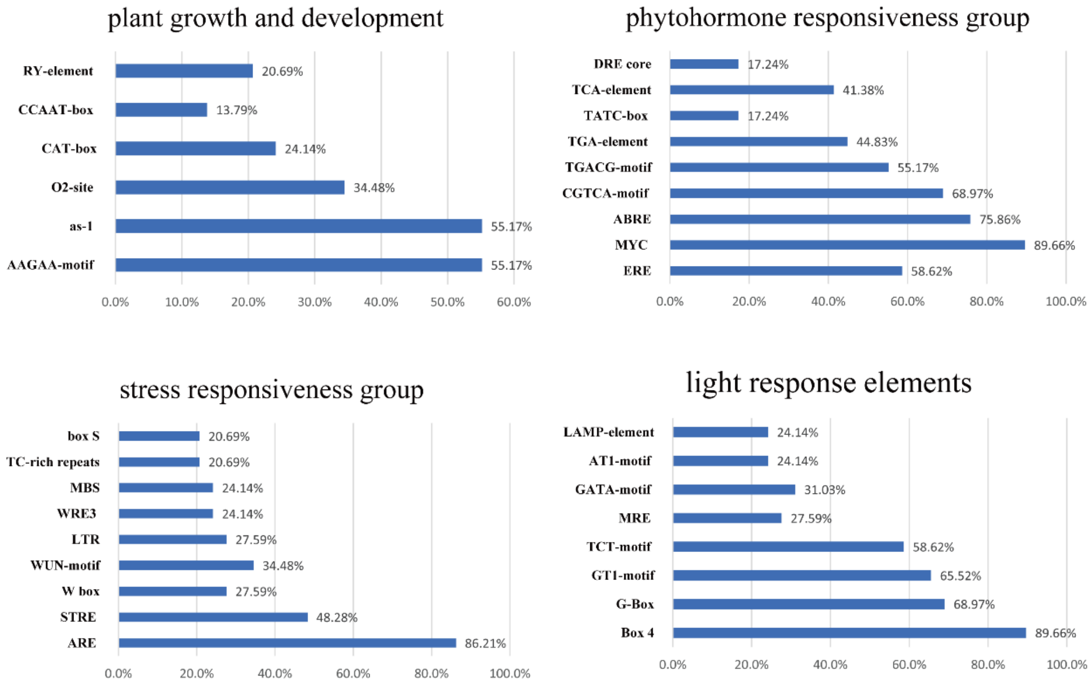

Figure S3. Four types of CREs, which are related to light reponsive element, plant growth development, phytohormone responsiveness and stress responsiveness, were predicted in the 2000-bp promoters of the 29 PyunKTI genes.
